# Effects of gender, activity type, class location and class composition on physical activity levels experienced during physical education classes in British secondary schools: a pilot cross-sectional study

**DOI:** 10.1186/s12889-020-09698-y

**Published:** 2020-10-21

**Authors:** Anne Delextrat, Patrick Esser, Nick Beale, Floris Bozon, Emma Eldridge, Hooshang Izadi, Heidi Johansen-Berg, Catherine Wheatley, Helen Dawes

**Affiliations:** 1 Centre for Movement, Occupational and Rehabilitation Sciences (MOReS), Oxford Brookes University, Headington Campus, Oxford, OX3 0BP UK; 2 Wellcome Centre for Integrative Neuroscience, FMRIB Centre, Nuffield Department of Clinical Neurosciences, University of Oxford, John Radcliffe Hospital, Oxford, OX3 9DU UK

**Keywords:** Multilevel statistical model, Vigorous physical activity, MVPA, Accelerometers

## Abstract

**Background:**

Pupils in secondary schools do not meet the targets for physical activity levels during physical education (PE) sessions, and there is a lack of data on the vigorous physical activity domain (VPA) in PE known to be positively associated with cardio metabolic health While PE session intensity depends on a variety of factors, the large majority of studies investigating these factors have not taken into account the nested structure of this type of data set. Therefore, the aim of this study was to investigate the relationship between various factors (gender, activity type, class location and class composition) and various activity levels during PE classes in secondary schools, using a multi-level statistical approach.

**Methods:**

Year eight (12–13 years old) adolescents (201 boys and 106 girls) from six schools were fitted with accelerometers during one PE session each, to determine the percentage (%) of the PE session time spent in sedentary (SPA), light (LPA), moderate (MPA), vigorous (VPA) and moderate-to-vigorous (MVPA) intensity levels. Two- and three-level (pupils, *n* = 307; classes, *n* = 13, schools, *n* = 6) mixed-effect models were used to assess the relationship between accelerometer-measured physical activity levels (% of class time spent in various activity levels) and gender, activity type, class location and composition.

**Results:**

Participants engaged in MVPA and VPA for 30.7 ± 1.2% and 11.5 ± 0.8% of PE classes, respectively. Overall, no significant association between gender or class composition and PA was shown. A significant relationship between activity type and PA was observed, with Artistic classes significantly less active than Fitness classes for VPA (5.4 ± 4.5 vs. 12.5 ± 7.1%, *p* = 0.043, *d*:1.19). We also found a significant association between class location and PA, with significantly less time spent in SPA (24.8 ± 4.8% vs. 30.0 ± 3.4%, *p* = 0.042, *d*:0.77) and significantly more time spent in VPA (12.4 ± 3.7% vs. 7.6 ± 2.0%, *p* = 0.022, *d*:1.93) and MVPA (32.3 ± 6.7% vs.24.8 ± 3.8%, *p* = 0.024, *d*:1.33) in outdoors vs. indoors classes.

**Conclusions:**

The results suggest that class location and activity type could be associated with the intensity of PA in PE. It is essential to take into account the clustered nature of this type of data in similar studies if the sample size allows it.

## Background

Children and adolescents who engage in moderate-to-vigorous physical activity (MVPA) are at lower risk of developing chronic health issues, such as cardiovascular, metabolic or mental health issues [1–3]. In particular, the vigorous PA domain (VPA) has been associated with a more favourable cardiometabolic risk profile in youth [4], and with greater benefits than moderate physical activity (MPA) on adiposity, bone health, insulin and blood lipid profiles, and blood pressure [4–6].

Despite these positive observations, physical activity trends globally indicate that only about 20% of adolescents meet the recommendation of the World Health Organisation (WHO) to participate in 60 min of MVPA daily [7]. Scientific evidence supports that most PA is undertaken as part of the curriculum during school time [8], highlighting the importance of school physical education (PE) and the need to optimise PA targets within compulsory PE lessons at school. In the United Kingdom (UK), the Association for Physical Education (afPE) suggests that, for young people aged 5–17, “pupils be actively moving for 50-80% of the available learning time”, or at least 50% of PE lesson time should be spent doing MVPA [9]. To our knowledge, no clear recommendation has been given yet in the UK regarding VPA in PE sessions. A recent meta-analysis in seven countries world-wide, including one study in the UK, reported that only 35.9% of high school PE classes time (pupils aged 14–18) was spent in MVPA [10]. Other studies on UK school children and adolescents, indicating the same trend (between 27 and 47% of PE time spent at MVPA in a review [11]) are more dated, highlighting the need for more recent data in this country. For VPA, a wide range of data is reported in the literature (from 5.1 to 34.8% of the PE class, [12–15]).

There is a variety of barriers to optimizing MVPA in PE sessions, such as institutional (school policies [16]), infrastructural (lack of/small sports halls [16]), pedagogical (class size, teaching approaches [17, 18]) or inter-individual factors (pupils’ motivation [19–21]). Amongst these factors affecting the amount of MVPA in PE lessons, gender is widely cited in the literature, with boys usually engaged in more MVPA than girls [12, 22–25]. This has been reported for VPA, but only by a few studies [12, 26], highlighting the need for additional data on VPA by gender during PE. Many reasons could explain this, including teaching style (more talking and explanations in girls classes, more active teaching style for boys), or psychological factors (girls refusing to sweat). However, it has also been suggested [12] that the extent of these observed gender differences couldbe skewed by the type of activities offered in PE sessions to boys and girls. Indeed, artistic activities (including gymnastics and dance) characterised by 30% of class time in MVPA, are more common in girls-only classes [27, 28] while ball games (including football, basketball, volleyball, handball and softball), with 48% of class duration spent doing MVPA, are more often found in boys-only classes [29, 30]. To our knowledge, only two studies reported VPA by activity type, showing a greater amount of VPA in fitness (athletic fitness, circuit, swimming) compared to ball games (football, basketball, rugby) and a lower amount of VPA in artistic activities (dance, gymnastics) compared to racket sports (tennis, badminton, table tennis), ball games and fitness [12, 13]. One factor that could be linked to activity type is lesson location. Indeed, more intense PE sessions are usually reported outdoors (41.4–45.7% of the time spent in MVPA) compared to indoors (33.7–36.1%)) [13, 31, 32], although no data is available on VPA. The greater MVPA outdoors has been attributed to larger spaces and a more favourable temperaturefor intense exercise [26]. Finally, gender differences in PA levels during PE have also been suggested to vary withclass composition, vizboys or girls only classes vs. mixed-gender (coeducation) classes [13, 26, 33–35]. These studies suggest that girls generally spend more time in MVPA when they are in mixed-gender classes [13, 26, 33, 34], possibly because they are motivated by boys’ greater activity levels, while no difference linked to class composition has been observed in boys [13, 34, 35]. The same has been observed for VPA, but only in one study [26].

While the literature is abundant on the association between various factors and PA levels during PE sessions, there are limitations that need to be addressed. There has been limited investigation into different activity domains, in addition to MVPA. In particular, VPA would be particularly interesting to examine in light of the significant health benefits associated with this domain, as described above [4, 6]. Furthermore, the conclusions drawn from data in most of the previous studies might be biased because they used a single level statistical analysis approach, where the data collected from each child is considered as independent from the others, while in reality it is not (children from the same class are not independent as they share the same teacher, for example). This has major implications for hypothesis testing, because it affects type 1 error and statistical power. Instead, using a contextual approach, such as multi-level modelling, seems more appropriate for this type of hierarchical data because it takes into account the variation in scores at different levels [36]. This statistical approach was used in very few similar studies on MVPA in PE classes [37–39].

Within this context, the main objective of the present study was to undertake a pilot study to investigate the relationship between various factors, including gender, activity type, class location and class composition, and the amount of VPA and MVPA during PE classes in secondary schools, whilst taking into account the influence of the specific classes and schools that these children belong to (i.e. exploring the variance explained by schools and classes). Two secondary objectives were to study the same relationships for other activity domains, including sedentary (SPA), light (LPA), moderate (MPA) and also to examine the interactions between these factors (gender*activity type, gender*location)in all the activity domains.

## Methods

### Participants

A convenience sample of 307 year-eight pupils (12–13 years old, 201 boys and 106 girls) was recruited from six schools and 13 classes. Participants and their parents were fully informed about the study procedure, and recruitment was performed on a parental opt-out basis, with a 100% response rate from schools and classes (no information was obtained about the reason for a pupil’s non-participation in a PE class). Eligibility criteria included being able to take part in PE sessions (i.e. no injury or illness preventing participation in physical activity). If a pupil returned the opt-out slip, they still participated in the PE lessons alongside their peers, but were not monitored. Gatekeeper approval was sought pre-study via the head teachers and opt-out consent was provided to both parents and children before the start of data collection; the study was approved by the Oxford University Central University Research Ethics Committee (ref # R48879/RE001). It was part of the ‘Fit-to-Study’ project funded by the Education Endowment Foundation and the Wellcome Trust (Grant Number 2681).

### Design and setting

This study used a cross-sectional design, with measurements taken for each participant during one PE class. Data were collected between September and December 2015 during a total of 13 PE classes randomly selected from six state schools within Oxfordshire (UK) which included five in an urban city, and one in a rural town location (https://onsdigital.github.io/postcode-lookup/). These schools were characterised by Indexes of Multiple Deprivation (IMD) ranging from 3.63 to 18.17, putting them in the first (two schools), second (three schools) and third (one school) quintiles. All the PE classes were taught by specialist PE teachers.

### Procedure

The dependent variables were the active PE session minutes (allocated time minus changing time pre and post) spent in various exercise intensity categories, including sedentary, moderate, VPA and MVPA. The time spent in the exercise categories was also scaled to minutes per hour, and expressed as a percentage (%) of the active PE session. The three main independent variables investigated are described in Table 1. They include gender, activity performed: ball games (including basketball, football, handball, netball and rugby) vs. artistic classes (including dance and gymnastics) vs. fitness classes (including boot camp and fitness) vs. racket sports (badminton), and class composition: boys-only, girls-only and mixed-gender classes.

**Table 1:** Descriptive data about the independent variables of the study

	Ball (***n*** = 146)	Artistic (***n*** = 54)	Fitness (***n*** = 52)	Racket (***n*** = 55)	Overall (***n*** = 307)
**Gender**					
Boy	124 (84.9%)	19 (35.2%)	13 (25.0%)	45 (81.8%)	201 (65.5%)
Girl	22 (15.1%)	35 (64.8%)	39 (75.0%)	10 (18.2%)	106 (34.5%)
**ClassType**					
Mixed	49 (33.6%)	35 (64.8%)	27 (51.9%)	27 (49.1%)	138 (45.0%)
Boys-only	97 (66.4%)	0 (0%)	0 (0%)	28 (50.9%)	125 (40.7%)
Girls-only	0 (0%)	19 (35.2%)	25 (48.1%)	0 (0%)	44 (14.3%)
**Location**					
Outdoors	122 (83.6%)	0 (0%)	27 (51.9%)	0 (0%)	149 (48.5%)
Indoors	24 (16.4%)	54 (100%)	25 (48.1%)	55 (100%)	158 (51.5%)

*n:* number of participants in each category

### Data processing

At the start of the class, each student was fitted with an accelerometer (Axivity AX3, Axivity Ltd., Melton Park, UK) incorporated in a silicon wristband, on their non-dominant wrist. This device is small (23 × 32.5 × 7.6 mm) and light (11 g) which did not interfere with the activities being performed whilst recording 3-axis accelerometry. Sampling frequency was set at 50 Hz (with a ± 8 g range) covering the allocated PE class time. Post PE-class, the raw accelerometry data were downloaded via the manufacturer’s software (version AX-GUI-28), after which they were processed, using 1 s epochs, via a bespoke LabView programme (National Instruments, Ireland). Data were selected according to active PE-class time as noted down via observation of the research team, which excluded time spent in changing rooms or awaiting instructions pre-start. Sample Frequency corrected cut-off levels, based on gravity subtracted single vector magnitude, were taken from Phillips et al. [40] from where sedentary (SPA), light (LPA), moderate (MPA) and vigorous (VPA) activity levels, expressed in minutes per hour, were derived. In addition, moderate to vigorous activity (MVPA) was calculated as the sum of moderate and vigorous activity.

### Statistical analyses

Statistical analyses were performed using R (R Core Team) [41], with lme4 [42]. Each dependent variable was presented as mean and standard deviation (Mean ± SD). A multilevel mixed-effects model with a three-level structure accounting for clustering at the school and class levels was first used (pupils *n* = 230, classes *n* = 13, schools *n* = 6), with a data transformation undertaken for the dependent variables using the Best Normalize function in R (URL https://github.com/petersonR/bestNormalize). This package uses the normalization function that best suits the data provided, amongst several such as Box-Cox transformation, the Yeo-Johnson transformation, Lambert WxF transformations, and the ordered quantile normalization transformation. Its aim is to make the data Gaussian and these transformations are reversible, so that any analysis performed on the normalized data can be interpreted using the original unit. However the model did not converge for schools and therefore a two-level structure was then adopted, with schools treated as a fixed effect and classes (nested within schools) as a random effect (random intercept). Differences between schools were analysed by a one-way analysis of variance (ANOVA), followed by a Tukey post-hoc test. At the lowest level of the model,the effect of gender, class composition, location and activity type, as well as the interaction between these factors, on the time spent doing SPA, MPA, VPA and MVPA during PE sessions were examined together in the model as fixed effects. Pairwise comparisons for the fixed factors (where model estimates indicated significance), were examined as differences of Least Squares (LS) Means adjusted according to Tukey. For all these analyses, a *p* value inferior to 0.05 was considered statistically significant. The proportion of the variance in each activity level accounted for by schools and classes was calculated by dividing the minimum variance quadratic unbiased estimators (MIVQUE) of each variance component by the sum of MIVQUEs. Effect sizes were calculated as Cohen’s *d (d*) and interpreted as small (0.2), medium (0.5), large (0.8) and very large (1.3) [43].

## Results

Overall, pupils spent 25.8 ± 1.4% of the active PE class in SPA, 19.2 ± 0.7% in MPA and 11.5 ± 0.8% in VPA activities. MVPA accounted for 30.7 ± 1.2% of the PE class. Significant differences between schools were shown for SPA (school 4 (18.6 ± 7.8 min) greater than 2 (14.4 ± 9.6 min) and 6 (13.3 ± 6.7 min), F: 3.358, *p* = 0.06, *d*; 0.48 and 0.73, respectively), VPA (schools all significantly different from each other, except 1 and 4 (4.4 ± 3.0 min and 4.3 ± 1.6 min), 2 and 3 (6.5 ± 4.3 min and 6.7 ± 4.6 min), and 5 and 6 (9.7 ± 5.0 and 9.8 ± 3.1 min), F:22.876, *p* = 0.0001, *d:* 0.62 to 2.23) and MVPA (schools 1 and 4 (15.2 ± 5.0 min and 16.0 ± 5.4 min) significantly lower than 5 and 6 (19.8 ± 8.1 min and 22.0 ± 4.4 min, *d*: 0.55 to 1.44).

### Statistical model

The dependent variables were examined at the ‘pupil’ level (lowest level of analysis), with the predictor variables being pupil gender, lesson location and lesson activity. The random effect terms (clustering) allowed for classes to be treated as random effects, but the model only converged when the school was treated as a ‘fixed’ effect (Model 1). A fully nested random effects model (with class nested in school) only converged for MVPA and VPA dependent variables (Model 2). In addition, the only fixed effect interaction that could be examined by both models was gender*activity (other interactions did not converge and hence could not be examined). Finally, the class composition variable did not converge for the SPA (Model 1), and thus its effects on the % of PE time spent in sedentary activities could not be analysed.

### Classes

Classes accounted for 9.7, 6.1 and 6.0% of the variance in the time spent in MPA, VPA and MVPA intensity domains (Model1), respectively.

### Gender

There was no significant relationship between gender and SPA (*p* = 0.374), MPA (*p* = 0.796), VPA (Model1: *p* = 0.729, Model2: *p* = 0.773), or MVPA (Model1&2: *p* = 0.712) levels during PE sessions (Table 2).

**Table 2:** Relationship between gender and the percentage of PE session time spent in various intensity domains (95% CI: 95% confidence intervals)

	Boy (***n*** = 201)	Girl (***n*** = 106)	Overall (***n*** = 307)	Effect sizes for comparisons
**Moderate and Vigorous Physical Activity (MVPA)**				
Mean (SD)	33.5 (10.4)	25.5 (9.96)	30.7 (11.0)	0.78
Median [Min, Max]	33.7 [10.2, 62.1]	24.0 [5.64, 48.5]	31.0 [5.64, 62.1]	
95% CI	32.1–34.9	23.5–27.4		
**Vigorous**				
Mean (SD)	12.7 (6.86)	9.39 (6.88)	11.5 (7.03)	0.47
Median [Min, Max]	12.4 [0.00, 29.4]	7.19 [0.606, 25.7]	9.78 [0.00, 29.4]	
95% CI	11.7–13.7	8.1–10.7		
**Moderate**				
Mean (SD)	20.9 (6.09)	16.1 (4.72)	19.2 (6.09)	0.85
Median [Min, Max]	21.1 [8.30, 41.7]	15.9 [3.64, 27.0]	19.0 [3.64, 41.7]	
95% CI	20.1–21.7	15.2–17.0		
**Light**				
Mean (SD)	43.5 (10.4)	43.8 (12.5)	43.6 (11.1)	0.03
Median [Min, Max]	42.4 [21.3, 75.2]	41.7 [15.8, 82.4]	42.0 [15.8, 82.4]	
95% CI	42.1–44.9	41.4–46.2		
**Sedentary**				
Mean (SD)	23.1 (10.5)	30.9 (14.2)	25.8 (12.4)	0.66
Median [Min, Max]	22.5 [3.55, 67.6]	29.1 [4.80, 77.9]	24.6 [3.55, 77.9]	
95% CI	21.6–24.6	28.2–33.6		

### Activity type

**Fig. 1:**
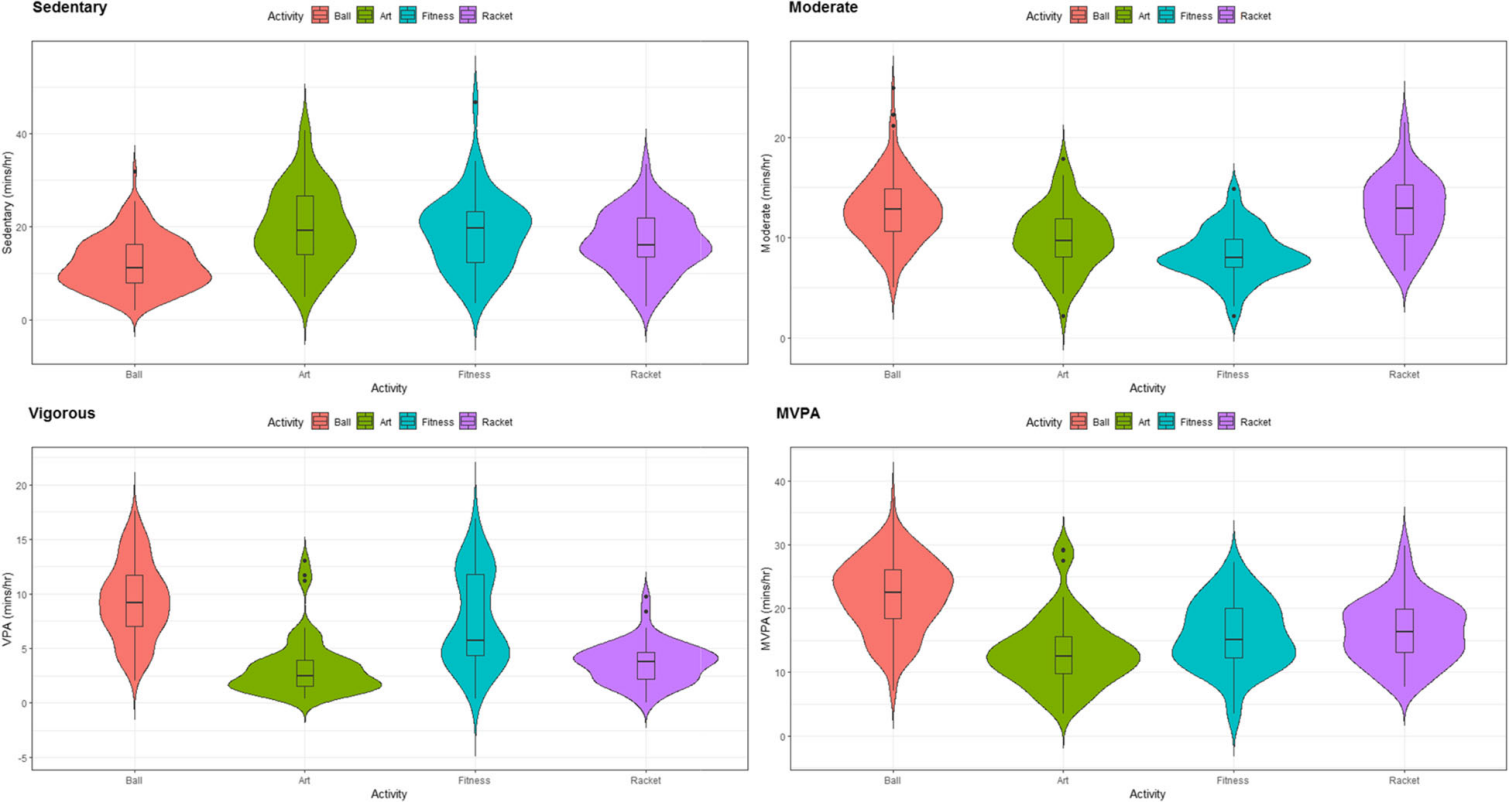
Effects of activity type on the PE session time spent in various intensity domains. MVPA: Moderate to vigorous physical activity

Significant associations between activity type and SPA and VPA were observed (Model1&2: *p* = 0.014 and 0.003, respectively), however the post-hoc comparisons (with “Tukey” pairwise adjustment) only showed a significant difference between Artistic (less time spent) and Fitness classes for VPA (*p* = 0.034); all other activity type and intensity domain combinations showed no significance (*p* = 0.073 to 0.998, Table 3 and Fig. 1).

**Table 3:** Relationship between activity type and the percentage of PE session time spent in various intensity domains

	A-Ball (***n*** = 146)	B-Artistic (***n*** = 54)	C-Fitness (***n*** = 52)	D-Racket (***n*** = 55)	Overall (***n*** = 307)	Effect sizes for comparisons
**Moderate and Vigorous Physical Activity (MVPA)**						
Mean (SD)	36.8 (9.60)	22.1 (9.21)	26.3 (8.91)	27.3 (8.23)	30.7 (11.0)	A-B: 1.5; A-C: 1.13; A-D: 1.06; B-C: 0.46; B-D: 0.60; C-D; 0.12
Median [Min, Max]	37.6 [11.7, 62.1]	20.8 [5.64, 48.5]	25.0 [6.06, 45.6]	27.2 [12.4, 49.8]	31.0 [5.64, 62.1]	
**Vigorous**						
Mean (SD)	15.5 (5.87)	5.43 (4.45)	12.5 (7.08)	6.10 (3.11)	11.5 (7.03)	A-B: 1.8; A-C: 0.46; A-D: 2.0; B-C: 1.19; B-D: 0.18; C-D; 1.18
Median [Min, Max]	15.3 [3.40, 29.4]	4.14 [0.727, 21.7]	9.51 [0.606, 28.1]	6.40 [0.00, 16.3]	9.78 [0.00, 29.4]	
**Moderate**						
Mean (SD)	21.3 (5.59)	16.6 (5.32)	13.9 (4.20)	21.2 (5.62)	19.2 (6.09)	A-B: 0.85; A-C: 1.50; A-D: 0.02; B-C: 0.56; B-D: 0.84; C-D; 1.47
Median [Min, Max]	21.3 [8.30, 41.7]	16.1 [3.64, 29.8]	13.3 [3.64, 24.8]	21.5 [11.2, 35.8]	19.0 [3.64, 41.7]	
**Light**						
Mean (SD)	43.0 (10.2)	45.0 (13.7)	42.5 (10.8)	45.1 (10.9)	43.6 (11.1)	A-B: 0.18; A-C: 0.05; A-D: 0.20; B-C: 0.20; B-D: 0.01; C-D; 0.24
Median [Min, Max]	42.0 [21.7, 71.1]	43.2 [20.9, 82.4]	40.8 [15.8, 70.9]	42.4 [25.3, 75.2]	42.0 [15.8, 82.4]	
**Sedentary**						
Mean (SD)	20.3 (9.30)	33.1 (13.8)	31.3 (13.5)	27.8 (10.9)	25.8 (12.4)	A-B: 1.20; A-C: 0.93; A-D: 0.74; B-C: 0.13; B-D: 0.43; C-D; 0.25
Median [Min, Max]	18.7 [3.55, 53.1]	31.8 [8.18, 67.6]	32.8 [5.93, 77.9]	26.8 [4.80, 55.6]	24.6 [3.55, 77.9]

### Interaction between gender and activity type

The statistical analysis did not show any significant interaction between gender and activity on SPA (*p* = 0.778), MPA (*p* = 0.825), VPA (Model1: *p* = 0.340, Model2: *p* = 0.296) or MVPA (Model1&2: *p* = 0.992).

### Class location

A significant relationship between class location and PA was shown, with outdoor classes characterised by significantly less time spent in SPA (24.8 ± 4.8% vs. 30.0 ± 3.4%, *p* = 0.042) and significantly more time spent in VPA (12.4 ± 3.7% vs. 7.6 ± 2.0%, *p* = 0.022) and MVPA (32.3 ± 6.7% vs.24.8 ± 3.8%, Model1&2: *p* = 0.024< Table 4).

**Table 4:** Relationship between location and the percentage of PE session time spent in various intensity domains

	Outdoors (***n*** = 149)	Indoors (***n*** = 158)	Overall (n = 307)	Effects sizes
**Moderate and Vigorous Physical Activity (MVPA)**				
Mean (SD)	37.0 (9.08)	24.8 (9.16)	30.7 (11.0)	1.33
Median [Min, Max]	37.1 [11.7, 62.1]	23.6 [5.64, 49.8]	31.0 [5.64, 62.1]	
**Vigorous**				
Mean (SD)	16.6 (5.72)	6.80 (4.35)	11.5 (7.03)	1.93
Median [Min, Max]	16.8 [3.40, 29.4]	6.40 [0.00, 21.7]	9.78 [0.00, 29.4]	
**Moderate**				
Mean (SD)	20.4 (6.08)	18.1 (5.90)	19.2 (6.09)	0.38
Median [Min, Max]	20.3 [7.41, 41.7]	17.8 [3.64, 35.8]	19.0 [3.64, 41.7]	
**Light**				
Mean (SD)	42.0 (10.0)	45.2 (11.9)	43.6 (11.1)	0.29
Median [Min, Max]	41.7 [21.7, 71.1]	42.6 [15.8, 82.4]	42.0 [15.8, 82.4]	
**Sedentary**				
Mean (SD)	21.2 (9.92)	30.1 (13.0)	25.8 (12.4)	0.77
Median [Min, Max]	19.6 [3.55, 53.1]	28.6 [4.80, 77.9]	24.6 [3.55, 77.9]	

### Class composition

A significant relationship between class composition and MPA (*p* = 0.028), VPA (Model1&2: *p* = 0.011) and MVPA (Model1&2: *p* = 0.034) activity levels was shown. However the post-hoc adjustments did not reveal any significant differences between boys-only, girls-only and mixed-gender classes for any intensity domain (*p* = 0.136 to 0.963, Table 5).

**Table 5:** Relationship between class composition and the percentage of PE session time spent in various intensity domains

	A-Mixed (***n*** = 138)	B-Boys-only (***n*** = 125)	C-Girls-only (***n*** = 44)	Overall (n = 307)	Effect sizes for comparisons
**Moderate and Vigorous Physical Activity (MVPA)**					
Mean (SD)	30.4 (9.97)	35.4 (10.0)	18.6 (6.06)	30.7 (11.0)	A-B: 0.50; A-C: 1.28; B-C: 1.84
Median [Min, Max]	30.9 [8.70, 49.1]	35.3 [11.7, 62.1]	18.3 [5.64, 32.4]	31.0 [5.64, 62.1]	
**Vigorous**					
Mean (SD)	12.4 (7.83)	12.8 (5.95)	5.25 (2.63)	11.5 (7.03)	A-B: 0.06; A-C: 1.00; B-C: 1.40
Median [Min, Max]	11.6 [0.00, 28.1]	12.4 [3.00, 29.4]	5.61 [0.606, 10.0]	9.78 [0.00, 29.4]	
**Moderate**					
Mean (SD)	18.1 (4.69)	22.6 (6.03)	13.4 (4.22)	19.2 (6.09)	A-B: 0.84; A-C: 1.03; B-C: 1.65
Median [Min, Max]	18.2 [7.39, 29.8]	22.7 [8.30, 41.7]	13.6 [3.64, 22.4]	19.0 [3.64, 41.7]	
**Light**					
Mean (SD)	42.4 (11.5)	44.6 (9.55)	45.1 (13.7)	43.6 (11.1)	A-B: 0.21; A-C: 0.22; B-C: 0.05
Median [Min, Max]	41.7 [21.3, 75.2]	42.9 [25.3, 71.1]	40.6 [15.8, 82.4]	42.0 [15.8, 82.4]	
**Sedentary**					
Mean (SD)	27.4 (11.7)	20.2 (9.01)	36.4 (14.8)	25.8 (12.4)	A-B: 0.69; A-C: 0.72; B-C: 1.56
Median [Min, Max]	26.5 [4.80, 67.6]	19.3 [3.55, 40.8]	36.1 [8.18, 77.9]	24.6 [3.55, 77.9]	

For all these results, model estimates are summarised in Table 6.

**Table 6:** Summary of physical activity in PE model estimates (β coefficient) for Model 1 (classes as random effects and schools as fixed effects) and Model 2 (fully nested random effects model), 95% confidence intervals (95% CI) and *p*-values

Model 1 Estimates	SPA		LPA		MPA		VPA		MVPA	
	β	95% CI	β	95% CI	β	95% CI	β	95% CI	β	95% CI
Girl^a^	0.33	−0.09 -0.74	0.03	−0.50 – 0.56	0.03	−0.41 – 0.46	0.04	− 0.32 – 0.40	0.05	− 0.36 – 0.46
Art^b^	0.44	− 0.07 –0.95	0.29	− 0.46 – 1.04	0.28	− 0.53 – 1.10	− 0.96***	−1.54 – − 0.39	− 0.4	−1.07 – 0.27
Fitness^b^	0.71*	0.19–1.23	− 0.11	− 0.80 – 0.59	−0.7	− 1.45 – 0.05	0.36	− 0.17 – 0.89	− 0.15	−0.77 – 0.46
Racket^b^	0.3	−0.10 –0.71	0	−0.59 – 0.58	0.45	−0.26 – 1.15	−0.75***	− 1.23 – − 0.28	−0.21	− 0.77 – 0.35
Indoors^c^	0.43*	0.10–0.76	0.21	−0.28 – 0.70	− 0.43	− 1.02 – 0.15	−0.54*	− 0.93 – − 0.14	−0.63*	− 1.09 – − 0.16
Girl:Art^d^	− 0.04	−0.69 –0.61	− 0.24	− 1.05 – 0.57	−0.24	− 0.91 – 0.43	0.16	− 0.38 – 0.71	−0.1	− 0.72 – 0.52
Girl:Fitness^d^	−0.3	− 1.05 –0.45	0.18	− 0.70 – 1.06	0.25	− 0.47 – 0.98	−0.4	−1.00 – 0.19	− 0.14	−0.81 – 0.53
Girl:Racket^d^	−0.32	−1.06 –0.42	0.37	−0.48 – 1.22	−0.01	− 0.73 – 0.71	−0.42	− 1.01 – 0.16	− 0.24	−0.90 – 0.42
Boys-only^e^			0.32	−0.08 – 0.73	0.65*	0.18–1.12	− 0.03	−0.36 – 0.29	0.26	−0.12 – 0.64
Girls-only^e^			0.11	−0.47 – 0.69	−0.32	− 0.97 – 0.33	−0.68*	−1.13 – − 0.23	−0.67*	−1.20 – − 0.14
Model 2 Estimates							VPA		MVPA	
							β	95% CI	β	95% CI
Girl^a^							0.05	−0.30 – 0.41	0.05	−0.36 – 0.46
Art^b^							−0.94**	−1.50 – − 0.38	−0.40	−1.07 – 0.27
Fitness^b^							0.34	−0.18 – − 0.92	−0.15	− 0.77 – 0.46
Racket^b^							−0.78**	−1.22 – − 0.35	−0.21	− 0.77 – 0.35
Indoors^c^							−0.47	− 0.90 – − 0.05	−0.63*	−1.09 – − 0.16
Girl:Art^d^							0.19	−0.36 – 0.74	−0.10	− 0.72 – 0.52
Girl:Fitness^d^							−0.46	−1.05 – 0.13	− 0.14	−0.81 – 0.53
Girl:Racket^d^							−0.43	−1.01 – 0.15	− 0.24	−0.90 – 0.42
Boys-only^e^							0.02	−0.30 – 0.33	0.26	−0.12 – 0.64
Girls-only^e^							−0.71*	−1.18 – − 0.23	−0.67*	− 1.20 – − 0.14

^a^ Reference category: Boys; ^b^ Reference category: Ball games^; c^ Reference category: Outdoors;

^d^ Reference category: Boys:Ball games; ^e^ Reference category: Co-education

*** *p* < 0.001; ** *p* < 0.01; * *p* < 0.05

## Discussion

This is a pilot study investigating, for the first time, the relationship between various factors including gender, activity type, class location and composition, and exercise intensity levels in secondary school students in the UK using a contextual statistical approach, partly taking into account the hierarchical structure of this type of data set. Our results should inform larger-scale cohort studies on the relevant data collection, and the main relationships and interactions to investigate, using a hierarchical structure. Our main findings indicated that year eight (12–13 years old) UKstudents did not achieve the afPE currently recommended levels of activity. In relation to our first two objectives, we found significant relationships between activity type and class location, with students engaging in higher intensity PA in Fitness classes (including fitness and bootcamp) compared to Artistic classes (including dance and gymnastics), and in outdoor classes compared to indoor classes. However, we did not observe any significant association between gender or class composition and PA. In relation to our third objective, no significant interaction between gender and activity type was shown. Our approach and findings, which in some aspects contradict other observations, perhaps highlight the complexity of measuring physical activity in school PE, and the importance of taking into consideration the impact of school and class in analytical approaches if researchers are to interpret data from schools and develop guidelines to inform teaching and health promotion practices.

Our results showed that British year eight students do not reach the recommendations by the afPE [9] to spend 50% of PE lessons time doing MVPA. MVPA accounted for just 30.7 ± 1.2% of the PE class. Also, only 3.3% (10/307) of pupils in this pilot study met the afPE recommended MVPA threshold. Other countries usually recommend similar thresholds (minimum 50% of PE time in MVPA [44]), with some authors suggesting a lower cut-off point (33% of the PE session in MVPA in elementary school [45]), which our participants did not meet either. These rather low activity levels mirror results from other recent studies worldwide, with 35.9% (range 28.3–43.6%) of the PE session in MVPA reported in a meta-analysis of 25 studies from seven countries in adolescents aged 12–18 years old [10], and 27 to 47% of PE class time spent in MVPA in another review of 40 studies [11]. These results could be explained by the other pedagogical goals of PE classes, including cognitive, motor, moral, spiritual, social and creative development, which could interfere with the maximisation of exercise intensity as they require substantial time where students are not active [11, 46, 47]. In the UK, this could be amplified by the implicit nature of the PE curriculum compared to other countries, since there is no guidance within the English National Curriculum for PE to inform teachers how to increase physical activity levels in PE sessions. Other barriers to the development of high-intensity PE classes could be described as institutional, with various school policies, limited facilities, or a crowded curriculum potentially resulting in a decreased interest and time allocated for PE compared to more traditional areas, such as science or literature [16]. Finally, other factors also play a crucial role, such as differences in pedagogical variables (class size, available space, organizational strategies, teaching approaches, lesson content, etc), [17, 18] or inter-individual factors, including teachers’ beliefs, skills and confidence, or students’ motivation, ethnicity, socio-economical status, interests and gender [19, 20, 48, 49].

When using a hierarchical analysis we found no significant relationship between gender and the proportion of PE session time spent in all activity level domains. In accordance with these findings, a few previous studies have also failed to observe any association between gender and PA levels during PE sessions in Portuguese and Swedish adolescents [14, 50]. However, the majority of studies report that boys engage in more VPA and MVPA than girls in PE [12, 22–26]. These studies are characterised by larger samples than ours, and hence our study might be slightly underpowered, partly explaining these contrasting results. However, it is also important to note at this point that none of these previous studies have used hierarchical statistical analysis,. In the context of the present study, our findings could be interpreted as a better ability of the PE sessions to engage girls in moderate and high-intensity activity compared to previous studies, butthe trend for lower MVPA and greater SPA suggests the need for further investigations on larger samples of school adolescents. Alternatively, our results could be explained by other factors including specific school policy, the type of teachers delivering the PE sessions (specialists) or different activity types offered to boys and girls in PE [16, 27–30].

We only found one significant association between activity type and PA. Students in Artistic PE sessions, including dance and gymnastics engaged in significantly less VPA than Fitness sessions, including fitness and bootcamp), with a large effect size (*d*: 1.19). This large effect size suggests that these differences are practically meaningful, and would translate into around 9 min more VPA in Fitness classes than Artistic classes per week, for a class with 2-h of PE per week. This is in accordance with two studies [12, 13], however the lack of data on VPA in the literature suggests the need for more studies in this area. Our results on MVPA are in disagreement with most past studies [11, 29, 51, 52], showing more time spent at this intensity when ball games (including football, softball, basketball, netball and handball) were the focus of the session compared to other activity types. Regarding artistic class (dance and gymnastics), difficulty in reaching higher levels of intensity (VPA) in this type of activity compared to lower intensity levels (MPA, LPA, SPA) could be a plausible explanation. However, it should be noted that, again these past studies did not take into account the nested structure of this type of data (participants nested in classes, as weaccounted for, classes nested in schools, that could not be taken into account), and therefore it should be highlighted that previous literature may have been influencedby this approach. As mentioned before, it is difficult to compare VPA with existing literature (only two studies on activity type and VPA [12, 13]) because most studies only considered MVPA and did not break down activity levels into other intensity zones. The inclusion of several categories of physical activity in future studies would be essential to allow a better comparison of activity patterns in the literature and thus enhance our understanding of the demands of PE sessions. In particular, it has been suggested the advantages of VPA compared to MVPA thresholds are a greater association with future cardiovascular health [26], and a better differentiation between groups, as it is relatively easy to reach moderate vs vigorous levels of physical activity [30].

Our results did not reveal any significant interactions between gender and activity level, which is in agreement with the findings of Stratton et al. [30] and Kulinna et al. [29]. Conversely, Froberg et al. [50] showed a significant gender by activity type interaction, with boys engaged in significantly more MVPA than girls in ball games and dance activities. The discrepancy between these results and those of the present study could be due to the cut-offs used to define MVPA. Indeed, Froberg et al. [50] showed different results when using two different cut-offs [53, 54]. The absence of interaction reported in our study could be explained by students’ age. When contrasting results by age, Froberg et al. [50] found no significant differences between genders on MVPA in grade 5 and 8 (12 and 15 years old, respectively), and a significant gender effect in grade 2 (9 years old). They suggested that there was a good opportunity to reach girls between 12 and 15 years old and motivate them to be more active during PE sessions.

It has been suggested that lesson location influences the intensity of PE lessons [13, 26, 31]. In accordance with these studies, we observed that outdoor classes led to a greater proportion of MVPA and VPA compared to indoor classes, and less time spent in SPA. There were moderate to large effect sizes associated with these differences, suggesting the importance of lesson location in a practical context and the potential benefits of using outdoor classes in the aim of increasing the intensity of a PE session. Similarly, in the literature more intense PE sessions are usually reported outdoors (41.4–45.7% of the time spent in MVPA) compared to indoors (33.7–36.1% of the time spent in MVPA), [13, 31], although no data is available in the literature on lesson location and VPA. In addition, Chow et al. [26], used multiple regressions and showed that the proportion of PE sessions allocated to MVPA was positively linked to the size of the class area and negatively associated with air temperature. These factors could explain our results, in particular as our study was undertaken in winter in the UK While these results could point to outdoor PE classes being prioritised to achieve greater intensity levels, the implication of our findings is limited by several factors. First, ‘class’ does not explain a large proportion of the variance of the time spent in any activity level (up to 9.0%). Second, the various activity types are not proportionally represented in the outdoors and indoors sessions (for example 83.6% of ball sports are performed outdoors and 100% of artistic classes are indoors), which can bias findings. Indeed, looking at the data in Tables 3 and 4, the amount of time in MVPA and VPA match closely between Outdoors and Ball sports (37% vs. 36.8% of MVPA and 16.6% vs. 15.5% of VPA, respectively for Outdoors and Ball sports), as well as between Indoors and Racket Sport (24.8% vs. 27.3% of MVPA and 6.8% vs. 6.1% of VPA, respectively for Indoors and Racket sports). While this suggests that knowing that rugby was played outdoors makes it a more intense activity beyond the fact that rugby was played, larger data sets with a better representation of activity types are necessary to confirm these findings.

A crucial factor cited in the literature to account for differences in activity levels during PE classes is class composition, with past studies indicating that girls experienced more MVPA [13, 26, 31, 33, 34] and VPA [21] in mixed-gender classes compared to girls-only classes. In these studies, no difference was reported between boys-only and mixed gender classes [13, 34, 35]. where less time was spent giving instructions in boys-only classes, and that instructions were often given while the students were standing up, while teachers tend to give instructions to girls while they are seated [26, 34]. While it is difficult to compare our data on girls with the literature due to a limited amount of girls-only classes, our findings are in agreement with past studies on boys. It is interesting to note that the absence of significant relationship between class composition in boys previously reported remains even after taking into account the nested structure of this type of data. However our data, as well as those from previous studies, could be biased by the different types of activities used in mixed-gender vs. boys only classes. To our knowledge, no study has previously addressed this issue, and unfortunately we could not statistically account for the interaction between these factors due to the lack of convergence of the class composition data in our model, suggesting high levels of variability. However if we examine lesson content in our study, as presented in Table 1, we can observe that ball games (football, rugby, basketball, netball, handball) were represented at 33.6% in our data in mixed-gender classes compared to 66.4% in the boys-only classes.

The main limitations of this study are the relatively small sample size, which did not enable a fully nested random structure (school, class) to converge with all fixed-effect terms of interest included. The lack of data on the schools/classes/pupils (ethnicity, information about teachers and reasons for pupils non-participation in PE class) and the fact that we only recorded PA during one PE class per student should also be acknowledged as potential confounding factors and the cross-sectional nature of our design does not allow causal conclusions. Our tool to measure PA, accelerometry is also associated with potential limitations, such as imprecision tin quantifying heavy work done isometrically, and the variability in PA levels presented between studies using different cut-offs [50]. However, it should be pointed out that there is currently no gold standard in PA measurements, and other tools are also characterized by limitations (inter-rater reliability issues of the System of Observing Fitness Instruction Time (SOFIT), vulnerability of physiological factors, such as heart rate to parameters other than PA levels, such as stress or emotions). Finally, while the main strength of our statistical approach was to account for the hierarchical structure of the data, it was only possible to apply this model for the MPA and VPA intensity domains. It was also not possible to examine all the independent variables (e.g. class composition in SPA) and/or interactions (only one possible was gender*activity type) due to a lack of convergence of the data. To address this issue, the data set should include a better representation of each sub-type of independent variable in each class/school. However, this cannot be forced if the aim of the study is to examine current practices without intervening. It is possible that further studies with larger sample sizes will partly address this issue and provide some insight into these interactions.

## Conclusions

In conclusion, our results suggest that class location (classes located outdoors lead to the greatest MVPA and VPA) and the choice of activity (for example we found that Artistic classes such as dance and gymnastics were significantly less active than Fitness-based classes, such as fitness and bootcamp) may affect the intensity of PA in PE, and it would be important to consider these aspects when planning PE sessions. Considering the importance of school in PA of young people, further studies taking into account the nested structure of this type of data set are needed on larger samples to address the increasing concerns of PA levels of adolescents. One such project is the ‘Fit to Study’ trial (10.1186/s13063-019-3279-6). This is looking to advance the understanding of the complex relationships between school PE, PA and aerobic fitness, and whether school PE is an effective setting to increase the PA and fitness levels of adolescents.

## Data Availability

The datasets used and/or analysed during the current study are available from the corresponding author on reasonable request.

## Abbreviations

PE: Physical eduation
PA: Physical activity
SPA: Sedentary physical activity
LPA: Light physical activity
MPA: Moderate physical activity
VPA: Vigorous physical activity
MVPA: Moderate-to-vigorous physical activity
WHO: World Health Organisation
UK: United Kingdom
afPE: Association for physical education
IMD: Indexes of multiple deprivation
ANOVA: Analysis of variance
LS: Least squares
MIVQUE: Minimum variance quadratic unbiased estimators
*d*: Cohen’s d
95% CI: 95% confidence intervals
